# Anaphylaxis in a Swiss university emergency department: clinical characteristics and supposed triggers

**DOI:** 10.1186/s13223-024-00901-y

**Published:** 2024-05-31

**Authors:** Simone Ehrhard, Vicky Eyb, Dominic Gautschi, Stefan K. Schauber, Meret E. Ricklin, Jolanta Klukowska-Rötzler, Aristomenis K. Exadaktylos, Arthur Helbling

**Affiliations:** 1 Department of Emergency Medicine, Inselspital, Bern University Hospital, University of Bern, Freiburgstrasse 16C, 3010 Bern, Switzerland; 2 Centre for Health Science Education, Faculty of Medicine, University of Oslo, Oslo, Norway; 3 Division of Allergology and Clinical Immunology, Department of Pulmonary Medicine and Allergology, Inselspital, Bern University Hospital, University of Bern, Freiburgstrasse 16, 3010 Bern, Switzerland

**Keywords:** Anaphylaxis, Allergic reaction, Symptoms, Triggers, Emergency medicine

## Abstract

**Background:**

Anaphylaxis is the most severe form of acute systemic and potentially life-threatening reactions triggered by mast and basophilic cells. Recent studies show a worldwide incidence between 50 and 112 occurrences per 100,000 person-years. The most identified triggers are food, medications, and insect venoms. We aimed to analyze triggers and clinical symptoms of patients presenting to a Swiss university emergency department for adults.

**Methods:**

Six-year retrospective analysis (01/2013 to 12/2018) of all patients (> 16 years of age) admitted with moderate or severe anaphylaxis (classification of Ring and Messmer ≥ 2) to the emergency department. Patient and clinical data were extracted from the electronic medical database of the emergency department.

**Results:**

Of the 531 includes patients, 53.3% were female, the median age was 38 [IQR 26–51] years. The most common suspected triggers were medications (31.8%), food (25.6%), and insect stings (17.1%). Organ manifestations varied among the different suspected triggers: for medications, 90.5% of the patients had skin symptoms, followed by respiratory (62.7%), cardiovascular (44.4%) and gastrointestinal symptoms (33.7%); for food, gastrointestinal symptoms (39.7%) were more frequent than cardiovascular symptoms (36.8%) and for insect stings cardiovascular symptoms were apparent in 63.8% of the cases.

**Conclusions:**

Average annual incidence of moderate to severe anaphylaxis during the 6-year period in subjects > 16 years of age was 10.67 per 100,000 inhabitants. Medications (antibiotics, NSAID and radiocontrast agents) were the most frequently suspected triggers. Anaphylaxis due to insect stings was more frequently than in other studies. Regarding clinical symptoms, gastrointestinal symptoms need to be better considered, especially that initial treatment with epinephrine is not delayed.

## Background

Anaphylaxis represents the most severe clinical condition of an acute systemic reaction comprising two or more organ systems due to the activation of mast cells, basophils, and other immune cells with release of various mediators [1, 2]. Recent studies show a worldwide incidence between 50 and 112 occurrences per 100,000 person-years, while the estimated lifetime prevalence is 0.3–5.1% [3–6]. However, the mortality rate remains low, estimated at 0.05 to 0.51 per million people per year for medications, 0.03 to 0.32 for food, and 0.09 to 0.13 for venom-induced anaphylaxis [7, 8]. The most frequently cited triggers for anaphylaxis are food, medications, and insect stings [8, 9]. While food is the most common trigger for anaphylaxis in children [10], medications and insect venoms are most common in adults [5]. Idiopathic anaphylaxis is present when no trigger can be identified; it accounts for between 6.5 and 35.0% of cases, depending on the population and study site [11].

Anaphylaxis is a clinical diagnosis, based on signs and symptoms that appear within minutes and < 2 h after exposure to an allergen or trigger [12]. In 2005, clinical criteria for the diagnosis of anaphylaxis were proposed at the second US National Institute of Allergy and Anaphylaxis Network (NIAID/FAAN) symposium [13], which have been simplified to the current NIAID/FAAN criteria: first, typical skin symptoms AND significant symptoms in at least one other organ system (including gastrointestinal, respiratory, and cardiovascular); OR second, exposure to a known or probable allergen for that patient with respiratory and/or cardiovascular impairment [1, 14]. Nevertheless, the spectrum of possible clinical symptoms is broad, sometimes skin symptoms are absent or symptoms are nonspecific, and evaluation of clinical course and diagnosis can be difficult which may delay appropriate treatment [13, 15–17]. Thus, primary care physicians and emergency medical services have a crucial role in the prompt recognition and treatment of patients with anaphylaxis as this may reduce morbidity and mortality [17].

The aim of this study is to evaluate suspected triggers and clinical symptoms of patients with anaphylactic reaction and to provide an overview for general practitioners and emergency services.

## Methods

### Study design

This single-center retrospective cohort study was conducted at the Department of Emergency Medicine for Adults of the University Hospital, Inselspital, Bern, Switzerland. The study period lasted from 1 January 2013 to 31 December 2018. All patients aged ≥ 16 years who were treated for anaphylaxis at the emergency department (ED) were included.

### Search strategy and eligibility criteria

A full text keyword-search was performed in the diagnosis list (first or second diagnosis) of the medical reports of all patients admitted to our ED within the given time period using the following defined keyword list combined with the Boolean operator “OR “: allergic reaction, allergic shock, anaphylaxis and anaphylactic shock. Second, one experienced physician (VE) and one advanced pre-graduated medical student (DG) screened (one person per patient) the full text fields “diagnoses”, “history” and “clinical assessment” for characteristics of acute anaphylaxis and classified the severity of anaphylaxis for each patient according to the criteria of Ring and Messmer [18]. As present anaphylaxis criteria are typical skin symptoms AND significant symptoms from at least 1 other organ [1], patients with only skin symptoms were excluded after the above-mentioned grading process. Patients without acute anaphylaxis as reason for admission and patients who refused or later withdrew their general consent for the use of their anonymized data were excluded from the study.

### Data collection and extraction

Data were extracted from the database of the patient management system of the Department of Emergency Medicine for Adults of the Inselspital, Bern University Hospital, Switzerland (Ecare, Turnhout, Belgium). Two persons (VE, DG) carried out the coding of the data that was not automatically extracted from the electronic patient file. Coding was performed using a coding book and after a training phase. The coding was supervised by the investigator (SE):

i) Demographic data (sex, age), vital parameters, triage data (the initial triage at our ED is routinely performed for every patient by specially trained nurses according to the Swiss Triage Scale) [19]
ii) Data on comorbidities for each patient (i.e., previous allergic reaction / previous anaphylaxis and its suspected trigger, known asthma), extracted manually from the full ED report (VE, DG)
iii) Data on suspected triggers of the actual episode, extracted manually from the full ED report (VE, DG)
iv) Data on symptoms (i.e., skin / mucosal, gastrointestinal, respiratory and cardiovascular system), time interval after trigger contact and appearance of symptoms, time interval after symptom onset and ED presentation, extracted manually from the full ED report (VE, DG)

### Statistical analysis

The statistical analysis was performed with SPSS for windows version 25.0 (SPSS Inc, Chicago, IL) and R Language for Statistical Computing (R Core Team, 2020). For descriptive analysis, the distribution of continuous variables, such as age, were described with median and interquartile range (IQR) as most continuous variables were not normally distributed. The distribution of categorical data was described with the total number accompanied by percent. Differences between groups (gender; severity) were compared using chi-square tests. Since these analyses were exploratory, we did not adjust for multiple comparisons.

### Ethical considerations

The regional ethics committee of the Canton of Bern, Switzerland approved the study (KEK: 2019-02349).

## Results

### Demographics

**Fig. 1:**
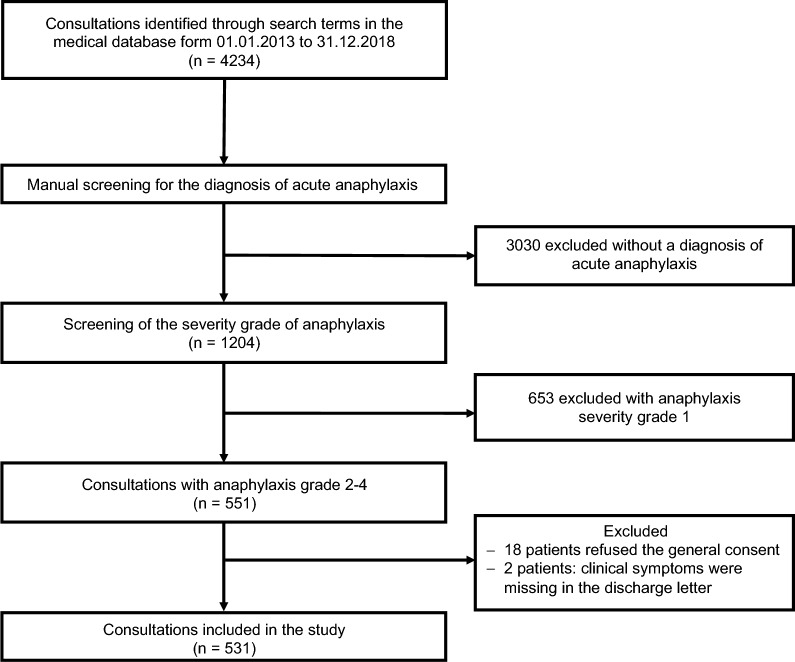
Patient flow chart

During the study period (1.1.2013–31.12.2018) 551 out of 260,485 patients referred to the ED department were identified in the medical database with diagnosis of anaphylaxis Grade II-IV according to the classification of Ring & Messmer. Eighteen patients refused to sign general consent, and detailed clinical symptom information was not available from two patients. Finally, 531 patients were included in the analysis (Fig. 1).

During the 6-year observation period, the average annual incidence of moderate and severe anaphylaxis was 10.67 per 100,000 inhabitants. 364 (68.5%) patients were classified as having grade II anaphylaxis, 162 (30.5%) were classified as having grade III anaphylaxis, and only 5 (0.9%) were classified as having grade IV anaphylaxis. The median age of the patients was 38 years (IQR 26–51), and 283 (53.3%) were female. Regarding the age distribution, 243 (45.8%) of the patients were ≤ 35 (16–35) years old. For grade 2 reactions, 92 (25.3%) patients were ≤ 25 years of age, grade 3 reactions occurred most frequently in 26–35-year-old patients (n = 38, 23.5%), and 4/5 of patients with grade 4 reactions were > 45 years of age.

In the personal history, 314 (59.1%) patients reported some type of allergy, 58 (10.9%) had asthma, and 179 (33.7%) had previous anaphylaxis. Nearly two-thirds (n = 313, 58.9%) of patients self-presented to the ED, 158 (29.8%) were admitted by ambulance, and 28 (5.3%) patients who were for outpatient treatment at the hospital and developed anaphylaxis were referred to the ED. Of the patients admitted, 85 (16.0%) patients were classified as STS category 1 and 307 (57.8%) as STS category 2. Ninety-one (17.1%) patients were treated in the rescue bay, all other patients were treated in regular rooms of the ED (Table 1).

**Table 1:** Demographic data, n = 531

	All patients		Female		Male		p-value
	n = 531	(%)	n = 283	%	n = 248	%	
*Severity grade of anaphylaxis*							0.114
Grade II	364	(68.5)	205	(72.4)	159	(68.5)	
Grade III	162	(30.5)	76	(26.9)	86	(34.7)	
Grade IV	5	(0.9)	2	(0.7)	3	(1.2)	
Age median (IQR)	38	(26–51)	36	25.5–50	38.5	(26–52.3)	
*Age group (years)*							0.227
16–25	128	(24.1)	71	25.1	57	23.0	
26–35	115	(21.7)	69	24.4	46	18.5	
36–45	98	(18.5)	50	17.7	48	19.4	
46–55	91	(17.1)	43	15.2	48	19.4	
56–65	53	(10.0)	25	8.8	28	11.3	
66–75	26	(4.9)	11	3.9	15	6.0	
≥ 76	20	(3.8)	14	4.9	6	2.4	
*History/comorbidities*							
Known allergy^1^	314	(59.1)	179	(63.3)	135	(54.4)	0.039
Aeroallergy^2^	134	(25.2)	77	(27.2)	57	(23.0)	0.263
Food allergy	127	(23.9)	73	(25.8)	54	(21.8)	0.279
Drug allergy	98	(18.5)	70	(24.7)	28	(11.3)	< 0.001
Hymenoptera venom allergy	48	(9.0)	21	(7.4)	27	(10.9)	0.165
Other allergies	35	(6.6)	23	(8.1)	12	(4.8)	0.128
Prior anaphylactic episode	179	(33.7)	94	(43.5)	85	(41.3)	0.639
Asthma	58	(10.9)	37	(13.1)	21	(8.5)	0.090
Refe*rral to ED*							0.453
Self-presentation	313	(58.9)	173	(61.1)	140	(56.5)	
By ambulance	158	(29.8)	81	(28.6)	77	(31.0)	
From physicians office	31	(5.8)	13	(4.6)	18	(7.3)	
Hospital internal	28	(5.3)	16	(5.7)	12	(4.8)	
By police	1	(0.2)	0		1	(0.4)	
*Triage category*^3^							0.093
Category 1	85	(16.0)	53	(18.7)	32	(12.9)	
Category 2	307	(57.8)	150	(53.0)	157	(63.3)	
Category 3	131	(24.7)	74	(26.1)	57	(23.0)	
Category 4	2	(0.4)	1	(0.4)	1	(0.4)	
Unknown	6	(1.1)	5	(1.8)	1	(0.4)	
*Place of treatment at the ED*							
Rescue Bay	91	(17.1)	47	(16.6)	44	(17.7)	0.729
*Length of ED stay (hours)*							0.894
< 1	15	(2.8)	6	(2.1)	9	(2.8)	
1–3	68	(12.8)	35	(12.4)	33	(12.8)	
3–6	211	(39.7)	113	(39.9)	98	(39.7)	
6–9	121	(22.8)	64	(22.6)	57	(22.8)	
> 9	113	(21.3)	63	(22.3)	50	(21.3)	
Unknown	3	(0.6)	2	(0.7)	1	(0.6)	

^1^Many patients had more than one known allergy

^2^Aeroallergens e.g., pollen, house dust mites, animal epithelia, fungal spores

^3^Swiss Triage System (STS), categories: 1 = treatment immediately by a physician, 2 = treatment within 20 min by a physician, 3 = treatment within 120 min by a physician, 4 = no urgent treatment situation

This table was partially published in a previous publication [23]

### Suspected triggers of anaphylaxis

**Fig. 2:**
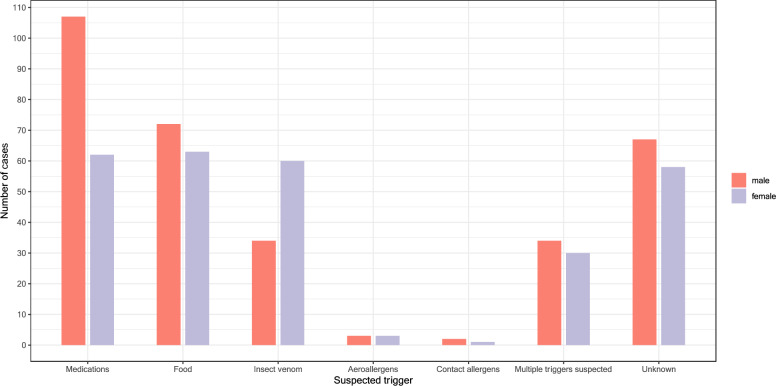
Suspected triggers: different frequency between women and men, n = 531

The most common suspected source was medications (n = 169, 31.8%), followed by food (n = 136, 25.6%), and insect stings (n = 94, 17.1%). Among drug-induced anaphylactic reactions, antibiotics (n = 44, 26.0%) and radiocontrast agents (n = 32, 18.9%) were the most frequently suspected triggers, followed by nonsteroidal anti-inflammatory drugs (NSAIDs) (n = 26, 15.4%), and 9 (5.3%) patients presented to the ED with a more severe allergic reaction after allergen-specific immunotherapy. Among food, tree nuts were the most frequent triggers (n = 28, 20.6%), followed by fruits (n = 13, 9.6%), shellfish (n = 12, 8.8%), and peanut (n = 8, 5.9%). In about a quarter of the cases, the causative trigger remained unclear (n = 125, 23.5%), and in 64 (12.1%) cases, multiple triggers were suspected (Table 2, Fig. 2).

**Table 2:** Suspected triggers of anaphylaxis, n = 531

Suspected triggers	All patients n = 531	
	n	%
Medications	169	31.8
Antibiotics	44	26.0
Penicillines	17	38.6
Quinolones	11	25.0
Cephalosporine	5	11.4
Clindamycine	1	2.3
Others	10	22.7
Radiocontrast agents	32	18.9
NSAID	26	15.4
Allergen-specific immunotherapy	9	5.3
Opioids	7	4.1
Others^1^	66	39.1
Food	136	25.6
Treenut	28	20.6
Fruits^2^	13	9.6
Crustacea	12	8.8
Peanut	8	5.9
Spices^3^	7	5.1
Wheat	6	4.4
Fish	6	4.4
Milk	5	3.7
Soya	5	3.7
Celery	5	3.7
Egg	4	2.9
Other^4^	8	5.9
Unclear food or more than one food possible^5^	40	29.4
Insect stings	94	17.7
Aeroallergens	6	1.1
Contact allergen	3	0.6
Multiple	64	12.1
Not determinable	125	23.5

In some patients, multiple allergens were suspected; therefore, the total number of all patients per trigger categories exceeds the total number of patients. The percentages refer to the respective category of suspected triggers

^1^Various medications

^2^Fruits including ananas (3x), kiwi (2x), strawberry (1x), banana (1x), mandarine (1x), orange (1x), melon (1x), cherry (1x), Yackfruit (1x), fruit juice (1x)

^3^Spices including curry (3x), curcuma (3x), paprica (1x)

^4^Other including almonds (2x), carrots (2x), mushrooms (2x), sage (1x), sesame (1x)

^5^In the unclear cases, no exact food trigger or a combination of food triggers were suspected, for example, a meal was eaten with subsequent allergic reaction

### Clinical manifestations

Onset of symptoms was significantly correlated with severity of anaphylaxis (p = 0.011).

Skin symptoms were noted in 471 (88.7%) patients, respiratory symptoms in 373 (70.2%), cardiovascular symptoms in 245 (46.1%), and gastrointestinal symptoms in 165 (31.1%) (Table 3).

**Table 3:** Organ systems^1^ involved in anaphylaxis

	n	(%)
Integument	471	(88.7)
Angioedema	236	(44.4)
Urticaria	192	(36.2)
Others (i.e., localized erythema)	216	(40.7)
Respiratory	373	(70.2)
Dyspnea/bronchospasm/wheezing	361	(68.0)
Worsening of established asthma	34	(6.4)
Nasal blockage	20	(3.8)
Rhinorrhea	17	(3.2)
Others (i.e., cough, sore throat)	79	(14.9)
Cardiovascular system	245	(46.1)
Low blood pressure^2^	102	(19.2)
Tachycardia^3^	77	(14.5)
Syncope	53	(10.0)
Bradycardia^4^	7	(1.3)
Spontaneous urine/stool loss	6	(1.1)
Cardiac arrest	5	(0.9)
Others (i.e., dizziness, cold sweat, shivering, chest pain)	114	(21.5)
Gastrointestinal tract	165	(31.1)
Nausea	81	(15.3)
Vomiting	51	(9.6)
Abdominal pain	47	(8.9)
Diarrhea	31	(5.8)
Others (i.e., urge to defecate, strange taste in mouth)	2	(0.4)
Time to symptom onset after trigger contact, in minutes		
< 30 min	247	(46.5)
> 30 min and < 120 min	34	(6.4)
> 120 min	49	(9.2)
Unknown	196	(36.9)

^1^Patients may have more than one organ system involved

^2^Systolic blood pressure < 100 mmHg

^3^Heart rate > 100 bpm

^4^Heart rate < 50 bpm

**Fig. 3:**
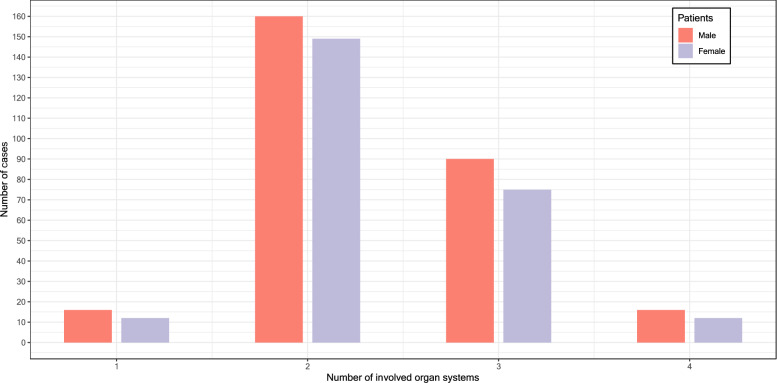
Number of affected organ systems, n = 531. In 29 patients, the one organ manifestation was interpreted in the context of anaphylaxis: gastrointestinal symptoms in 6 patients, respiratory symptoms in 16 patients, and cardiovascular symptoms in 7 patients

Referring to Ring and Messmer's classification, 58.2% of patients (n = 309) had two organ systems affected when referred to the ED. Skin plus respiratory symptoms (n = 173, 32.6%) were followed by skin plus cardiovascular systems (n = 61, 11.5%) and skin plus gastrointestinal symptoms (n = 49, 9.2%). Three organ systems were affected in 165 (31.1%) patients, and four organ systems were affected in 28 (5.3%) patients (Fig. 3).

Organ manifestations varied among triggers: when medications were suspected, skin symptoms occurred in 90.5% (n = 153), followed by respiratory symptoms (n = 106, 62.7%), cardiovascular (n = 75, 44.4%), and gastrointestinal symptoms (n = 57, 33.7). When food was suspected, gastrointestinal symptoms (n = 54, 39.7%) were more common than cardiovascular symptoms (n = 50, 36.8%). For insect bites, cardiovascular symptoms occurred in two-thirds of cases (n = 60, 63.8%). Respiratory symptoms occurred in all patients (n = 5, 100%) with reactions triggered by aeroallergens (Table 4).

**Table 4:** Organ systems affected with respect to suspected triggers

Triggers	Organ systems							
	Cutaneous symptoms	%	Gastrointestinal symptoms	%	Respiratory symptoms	%	Cardiovascular symptoms	%
Medications	153	90.5	57	33.7	106	62.7	75	44.4
Food	116	85.3	54	39.7	102	75.0	50	36.8
Insect stings	76	80.9	21	22.3	68	72.3	60	63.8
Aeroallergens	5	83.3	2	33.3	6	100	2	33.3
Contact allergens	3	100	1	33.3	2	66.7	1	33.3
Multiple triggers suspected	59	92.2	29	45.3	47	73.4	25	39.1
Unknown	120	96.0	31	24.8	89	71.2	54	43.2

## Discussion

The average annual incidence of moderate to severe anaphylaxis during the 6-year period at the University ED of Bern (Inselspital) was 10.67 per 100,000 inhabitants. This proportion, which is as frequent as emergency admission for ST-elevation myocardial infarction, is consistent with other study data on anaphylaxis in the ED (0.04 to 0.96% of all referrals) [5, 6, 20, 21] and is in the same range compared with a study conducted 20 years ago from the same catchment area, but with a lower proportion of severe anaphylaxis [22]. One explanation for this could be an increased awareness of allergic reactions, since one third of the patients stated that they already had anaphylactic reactions in the past. On the other hand, emergency medical services and perhaps patients have a clearer therapeutic strategy, especially with the use of epinephrine [23]. However, epidemiologic data on anaphylaxis should be interpreted with caution: First, because anaphylactic events often occur outside hospitals, so the true rate of anaphylaxis may be underestimated after all [8]. Second, direct comparisons with other studies are difficult, not least because of the use of different classifications of anaphylaxis.

In our collective, 2/3 were rated as moderate reactions and 1/3 as severe, of which 5 patients were in acute danger of death. Thus, 28 life-threatening anaphylaxis events can be expected per year in our ED. Since no one knows when the event will occur, it is important to be prepared. Therefore, continuous education in anaphylaxis management is important.

Two-thirds of patients with anaphylaxis were ≤ 45 years of age, whereas this diagnosis was only occasionally identified after the age of 65. In particular, for reactions to food over 50% of patients were younger than 35 years. This is consistent with food being the most common cause of anaphylaxis in children and adolescents [10]. That more severe systemic reactions decrease with age was also observed in a 3-year retrospective study in a Philippine ED hospital [24]. Based on data from various recording centers, the average age was generally between the 3rd and 5th decades of life, but a quarter of anaphylaxis occurred in persons younger than 18 years [20, 25, 26].

Two-thirds of our patient population had a known allergy, 10% had asthma, and one-third had previous anaphylaxis. Consistently with the literature, atopy is predominant in subjects with anaphylaxis [22, 24]. However, in terms of recurrences, the percentage of which may vary by reporting center and country, this underscores the importance of accurate allergy workup in affected individuals after diagnosis of anaphylaxis to prevent reoccurrences. Interestingly, almost 60% of the patients admitted directly and self to the ED. This may explain on the one hand the higher number of moderate systemic general reactions and on the other hand that the patients were sensitized to possible allergic reactions. It should also be taken into account that the hospital is centrally located in the city and thus easily accessible.

Medications were the most frequently suspected triggers for anaphylaxis, followed by food and insect stings. Although the percentage of anaphylaxis due to insect stings, 17.7%, may seem high compared with other studies of anaphylaxis [27, 28], this percentage was three times higher (58.8%) 20 years ago [22]. The large percentage difference between the two studies from the same region can be explained by the fact that the first study included all EDs in the canton of Bern and therefore a more rural population. However, the currently collected data is consistent with a previous study at our ED, which only analysed anaphylaxis associated with Hymenoptera stings [29].

Of the medications, antibiotics, radiocontrast agents, and NSAIDs were the most common suspected triggers of anaphylactic reactions. While antibiotics, especially penicillin and cephalosporin, and NSAIDs are known to be frequent triggers of anaphylaxis [15, 22], the occurrence of severe systemic reactions after administration of radiocontrast agents was so unexpected. This finding can probably be explained by the fact that the hospital is a tertiary center with many multimorbid patients who receive repeated radiocontrast agents for diagnostic reasons [30, 31]. Another interesting point is that about 2% of the collective with drug-induced anaphylaxis developed it immediately after allergen-specific immunotherapy. Whether additional cofactors were involved, was not investigated. Although allergen-specific immunotherapy is the only immunomodifying therapy for IgE-mediated aero- and hymenopteran venom allergies that induces tolerance, these reactions are iatrogenic [32].

It is noteworthy that no suspected cause was found in a quarter of the cases, although this is consistent with data from another study [24], but in contrast to previous results where about 5% from the same catchment area could not be explained [29]. Perhaps discipline has waned among patients or even primary care providers to find the causal cause of the allergic reaction. Reasons for this may be lack of time, lack of interest or fear of high financial costs. It is also interesting that some analyses have shown that previously confirmed triggers were not always the cause of the current anaphylaxis and that patients or even the emergency physician suspected otherwise [17].

Anaphylaxis is generally defined as an immediate systemic reaction involving two or more organ systems [20, 33]. While gastrointestinal symptoms are generally the least frequently recorded in diagnosed anaphylaxis, gastrointestinal symptoms were common and more frequent than cardiovascular symptoms in postulated food-induced anaphylaxis. This contrasts with a Polish study in which gastrointestinal symptoms were generally the least frequently documented symptoms, even when food was suspected [33]. Is gastrointestinal tract involvement, such as nausea, feeling sick, or abdominal pain, less regularly asked about in allergic reactions? According to international guidelines, gastrointestinal symptoms are eligible for therapy with intramuscular epinephrine for anaphylaxis [34]. In a previous study, we showed that especially patients with skin and gastrointestinal symptoms (anaphylaxis grade II) often do not receive the necessary therapy with epinephrine [23]. Therefore, when anaphylaxis is suspected, gastrointestinal symptoms must be actively sought.

### Limitations

The limitations of this study are consistent with retrospective studies using medical records as sole source for data. We cannot rule out documentation bias or missed patients, despite careful data extraction and analysis. There is a potential for misclassification bias as the severity grade of anaphylaxis was done retrospectively due to symptoms noted in the patient electronic data record. The diagnoses were made by the treating physicians at the ED, the coding was carried out by one person (VE, DG) and monitored by one person (SE). Furthermore, the results of the allergology follow-up, if performed, were not available for these analyses and only the information about the suspected triggers could be recorded.

## Conclusion

The degree of anaphylaxis ≥ 2 according to Ring & Messmer criteria was 9.18/100,000 population over a 6-year period in a tertiary hospital center. One third were classified as severe anaphylaxis, of which 5 patients were in acute danger of death. Per month, 2–3 life-threatening anaphylaxis events can be expected per year. Medications, followed by food and insect stings, were the most common suspected triggers. However, one quarter of the events were diagnosed as idiopathic. This number as well as the observation that 1/3 of the patients already had an anaphylactic reaction before, indicates that an allergological clarification after anaphylaxis is necessary. On the side of clinical manifestations, it is also important to specifically ask about gastrointestinal symptoms, even mild ones, so that initial treatment with epinephrine is not delayed.

## Data Availability

The datasets used and/or analysed during the current study are available from the corresponding author on reasonable request.

## Abbreviations

ED: Emergency department
IQR: Interquartile Range
KEK: Kantonale Ethikkommission
NIAID/FAAN: National Institute of Allergy and Anaphylaxis Network
NSAIDs: Nonsteroidal inflammatory drugs
STS: Swiss Triage Scale
